# Brain injury induces specific changes in the caecal microbiota of mice via altered autonomic activity and mucoprotein production

**DOI:** 10.1016/j.bbi.2016.04.003

**Published:** 2016-10

**Authors:** A. Houlden, M. Goldrick, D. Brough, E.S. Vizi, N. Lénárt, B. Martinecz, I.S. Roberts, A. Denes

**Affiliations:** a Faculty of Life Sciences, University of Manchester, Manchester, UK; b Laboratory of Neuroimmunology, Institute of Experimental Medicine, Budapest, Hungary; c Laboratory of Drug Research, Institute of Experimental Medicine, Hungarian Academy of Sciences, P.O.B. 67, H-1450 Budapest, Hungary; d Department of Pharmacology and Pharmacotherapy, Semmelweis University, Budapest, Hungary

**Keywords:** Stroke, Microbiota, Cerebral ischemia, Inflammation, Noradrenaline, Gut, Mucoprotein

## Abstract

Intestinal microbiota are critical for health with changes associated with diverse human diseases. Research suggests that altered intestinal microbiota can profoundly affect brain function. However, whether altering brain function directly affects the microbiota is unknown. Since it is currently unclear how brain injury induces clinical complications such as infections or paralytic ileus, key contributors to prolonged hospitalization and death post-stroke, we tested in mice the hypothesis that brain damage induced changes in the intestinal microbiota. Experimental stroke altered the composition of caecal microbiota, with specific changes in Peptococcaceae and Prevotellaceae correlating with the extent of injury. These effects are mediated by noradrenaline release from the autonomic nervous system with altered caecal mucoprotein production and goblet cell numbers. Traumatic brain injury also caused changes in the gut microbiota, confirming brain injury effects gut microbiota. Changes in intestinal microbiota after brain injury may affect recovery and treatment of patients should appreciate such changes.

## Highlights

• Ischaemic brain injury drives profound changes in the gut microbiota.
• The effects of brain injury on the gut microbiota include changes in goblet cells and noradrenaline.
• Traumatic brain injury (TBI) also changes the gut microbiota.

## 1 Introduction

It is becoming clear that intestinal microbiota play key roles in both host development and in maintaining homeostasis. Intestinal microbiota change with age and are also influenced by environmental factors such as diet, and disease (Flint et al., 2012, Wu, 2011, Spor et al., 2011). Recent research highlights the key role of microbial communities in the large intestine in essential immune defence mechanisms and control of inflammatory responses (Abt et al., 2012, Ganal et al., 2012). Impaired regulation of the intestinal microbiota is also known to contribute to diseases of the intestinal tract, and is linked with the development of diverse inflammatory conditions such as sepsis, metabolic disease or cancer (Ayres et al., 2012, Dupont and Dupont, 2011, Le Chatelier et al., 2013, Yoshimoto et al., 2013). Altered intestinal microbiota have also recently been linked to neuro-behavioural problems such as autism (Hsiao et al., 2013, Kang et al., 2013). In fact the effect of microbiota on brain function is profound with microbiota known to influence brain specific activity such as anxiety like behaviour, learning and memory, microglial activity and blood brain barrier integrity (Luczynski, 2016). However, much less is known about whether changes in the intestinal microbiota are themselves influenced by central nervous system function. Brain injury caused by stroke is the most common cause of lasting disability worldwide and has a huge socio-economic impact. Beyond the detrimental effects of the initial injury on outcome after acute cerebrovascular events, one of the key causes for death or prolonged hospitalization and impaired recovery of patients is the development of post-stroke infections (Dirnagl et al., 2007). In some clinical studies, preventive antibiotic therapy was found beneficial (Hetze et al., 2013), and recent data indicate that pattern recognition receptors that can recognise microbiota-derived products could contribute to stroke outcome (Denes et al., 2015, Caso et al., 2008). The enteric nervous system is under central autonomic control and it is believed that the autonomic nervous system contributes to regulation of intestinal immunity and microbiota (de Jonge, 2013). We and others have shown that acute brain injury induces diverse autonomic, neuroendocrine and inflammatory changes, which manifest in several organs in the body, leading to immunosuppression and the development of infectious complications (Dirnagl et al., 2007, Denes et al., 2010). The impact of an acute brain injury on the intestinal microbiota, and whether this is also influenced by neuroendocrine and inflammatory changes as a result of injury, is not known, but could be of importance to the outcome and recovery of the patient.

Here we tested the hypothesis that an acute brain injury induced by experimental stroke or traumatic brain injury would induce specific changes in the gut microbiota. We demonstrate that brain injury profoundly impacts on microbial communities in the caecum and that brain injury is associated with specific changes in microbiota. We propose that these changes are due to increased noradrenaline (NE) release from the autonomic nervous system into the gut. These changes may influence recovery after an acute brain injury.

## 2 Methods

### 2.1 Mice

Male 10–14 week-old C57BL/6 mice were kept at 22 °C ± 1 °C and 65% humidity with a 12 h light-dark cycle and had free access to food and water. All animal procedures were performed under appropriate project licence authority and adhered to the UK Animals (Scientific Procedures) Act (1986) and the Hungarian Act of Animal Care and Experimentation (1998; XXVIII, Section 243/1998), approved by the Animal Care and Use Committee of the IEM.

### 2.2 Middle cerebral artery occlusion (MCAo)

Transient focal cerebral ischemia was induced as described previously (Dénes et al., 2010). Briefly, mice were anaesthetised with isoflurane, the common carotid artery was exposed and cerebral ischemia was induced by an intraluminal filament that was advanced along the internal carotid artery, to occlude middle cerebral artery. After 60 min of MCAo, reperfusion was induced for 4 h or 72 h prior to sacrifice. Core body temperature was maintained at 37.0 °C ± 0.5 °C throughout the surgery by a heating blanket (Homeothermic Blanket Control Unit; Harvard Apparatus, Kent, UK) and monitored after recovery. After surgery, animals were returned to their cages and allowed free access to water and food. Neurological deficit in mice was assessed by using Bederson scores (4 point scale of increasing neurological deficit) as described previously (Dénes et al., 2010). Animals that showed no obvious neurological deficit at 4 h reperfusion after MCAo (score 1 at minimum) have been excluded from the studies pre hoc (n = 1).

### 2.3 Surgical controls

We investigated the effect of surgical manipulation and anaesthesia in the absence of experimental stroke on changes in the intestinal microbiota. To achieve this, two separate experimental conditions were used; the first involved only anaesthesia with no surgical manipulation, the second sham surgery, during which mice underwent all procedures as in the MCAo group, except for occlusion of the MCA with an intraluminal filament.

### 2.4 Traumatic brain injury (TBI)

A closed head model of TBI was performed in mice under isoflurane anaesthesia similarly to what has been described earlier, with slight modifications (Umschweif et al., 2014). After induction of anaesthesia, the skull was exposed by a small, midline longitudinal incision. The head was held in place and a plastic cone was placed on the skull 2 mm lateral of the midline after which a 100 g weight was allowed to fall on the top of the cone from a preestablished height resulting in injury to the left hemisphere, which localised around the affected cerebral cortex (Fig. 7). Mice were allowed to recover and 1 ml of saline was injected subcutaneously for rehydration. We used a relative mild form of TBI resulting in 2 out of 7 mice with substantial neurological deficit as assessed 72 h later. Sham animals were subjected to the same procedure except for head injury.

### 2.5 Pharmacological manipulation of the sympathetic nervous system

A group of mice was injected with the NE reuptake inhibitor atomoxetine (Sigma, 0.1 mg kg^−1^) and the α2-adrenergic receptor antagonist yohimbine (Sigma, 1 mg kg^−1^) administered intraperitoneally (0.2 ml/mouse in total), once daily for three subsequent days. Another group of mice received a single intraperitoneal injection of 6-hydroxydopamine (6-OHDA) (0.2 ml, Sigma, 100 mg kg^−1^) followed by 0.2 ml sterile saline for two subsequent days. Control mice were administered 0.2 ml saline daily for three days. Mice were sacrificed 72 h after the first injection; the caecum was quickly removed and was kept at −80 °C until use.

### 2.6 ELISA

To measure inflammatory changes and neurotransmitter levels in the gut, caecum tissues were washed in sterile saline and homogenised as described previously (Dénes et al., 2010). Samples were kept at −20 °C until processing. Protein concentrations calculated using a BCA assay (Pierce/Themo Fisher Scientific). Caecum homogenates were measured for granulocyte-colony stimulating factor (G-CSF), RANTES (CCL5), KC (CXCL1), MMP-9, interleukin 6 (IL-6), ICAM-1, VCAM-1 (R & D Systems, UK), adrenaline and noradrenaline (Eagle Biosciences, NH, USA), substance P (R & D Systems, UK), and serotonin (Enzo, UK) according to the manufacturers protocol.

### 2.7 DNA extraction

Genomic DNA was extracted directly from total caecal content (∼250 mg) using the QIAamp DNA Stool Mini Kit (Qiagen) with pathogen protocol.

### 2.8 Community profiling

Bacterial communities were profiled in the mouse caecum using Denaturing Gradient Gel Electrophoresis (DGGE), 454 sequencing (Roche, USA), and Illumina MiSeq (USA). DGGE assessment of the bacterial communities was as follows: PCR amplification of the 16S rRNA gene used universal primers 341F-GC and 518R (Muyzer et al., 1993), and reaction conditions: 5U BioTaq polymerase in 1X buffer (Bioline, UK), 1.5 mM MgCl_2,_ 20 pmol primers, 0.2 mM dNTPs, 5 μg BSA, and 10–50 ng of template DNA in a final volume of 50 μL. The cycle sequence consisted of initial denaturation step of 95 °C 5 min, then 30 cycles of 95 °C 1 min; 55 °C 1 min; 72 °C 1 min, and final extension of 72 °C 10 min. PCR products were purified (QiaGEN Minelute kit) before loading onto a DGGE gel (150 ng/lane). Samples were separated using the D-code system (Bio-Rad, USA) on 10% w/v acrylamide gel with a gradient of 30–70% denaturant at 60 °C for 16 h at 63 V. Gels were stained for 30 min using SYBR Gold (Invitrogen, USA). DGGE Gels were analysed with Phoretix 1D Advanced gel analysis software (Ver. 5.0, Nonlinear Dynamics Ltd.), with binary matrix of band presence/absence of individual bands used for sample comparison.

#### 2.8.1 Pyrosequencing

454 sequencing of the bacterial communities were as follows: The 16S rRNA gene was amplified using the modified 16S primers 66f and 518R (italicised) to include Lib-A (underlined) linker primer sequences required to 454 sequencing and a MID tag to allow sample pooling (Forward primer (Primer A-Key): 5′‐CGTATCGCCTCCCTCGCGCCATCAG(MID)*CAGGCCTAACACATGCAAGTC*‐3′.

Reverse primer (Primer B-Key): 5′‐CTATGCGCCTTGCCAGCCCGCTCAG*ATTACCGCGGCTGCTGG*‐3′.

Roche multiplex identifiers (MID tags), which are unique “barcode” sequences for each amplified sample were used to allow the pooling of different samples into the same sequencing run. Post sequencing these samples could then be separated on their MIDs back into the individual sample amplified for analysis. MIDs used are detailed in Table S1. All samples were amplified by PCR using the same batch of reagents/buffers to eliminate reagent difference effects, they were also amplified in triplicate to reduce variation in PCR amplicon products (Polz and Cavanaugh, 1998). Samples were amplified using the following reaction conditions: 3U Velocity polymerase in 1X buffer (Bioline, UK), 20 pmol primers, 0.2 mM dNTPs, and 10–50 ng of template DNA in a final volume of 50 μL. The cycle parameters used a low cycle number to reduce chimera production (Thompson et al., 2002), and were as follows: Initial denaturation 95 °C 2 min 30 s, then 18 cycles of 95 °C 10 s; 55 °C 10 s; 72 °C 30 s; with final extension of 72 °C 2 min. Triplicate PCR reactions were pooled, before size selection by gel extraction with the QIAquick gel extraction kit, concentrated by using MinElute PCR Purification Kit (Qiagen), quantified using Qubit dsDNA HS Assay (Life technologies) before pooling of MID tagged products in equimolar amounts in preparation for multiplex barcode pyrosequencing. Roche 454 GS-FLX sequencing was undertaken at the Centre for Genome Research, University of Liverpool. yielding a total of 305,216 reads Sequences were processed with flows trimmed to 400 and processed/cleaned using PyroDist for distance calculation, FCluster for clustering analysis, PyroNoise in mothur based on AmpliconNoiseV1.25 (Quince et al., 2011).

#### 2.8.2 Ilumina MiSeq

Sequencing of the bacterial communities were undertaken by Centre for Genome Research, University of Liverpool using paired end Ilumina MiSeq, and library prep was as follows: The 16S rRNA gene was amplified using the primers described by Caporaso et al. (2011) producing 254 bp insert, and then nested PCR to add MID tags and including Ilumina adaptor sequences as in the Ilumina *Nextera* protocol. Samples were amplified using the following reaction conditions: 1X Kapa Mastermix (KapaBiosystems, UK) 10 μM primers, and 5–10 ng of template DNA in 20 μl final volume. Samples were then purified using Ampure beads and resuspend in 10 μl. In the second round PCR 5 ng of template DNA is replaced with 9 μl of purified PCR product. The cycle parameters used a low cycle number to reduce chimera production (Thompson et al., 2002), and were as follows for both rounds of PCR: Initial denaturation 98 °C 2 min, then 10 cycles of 95 °C 20 s; 65 °C 15 s; 70 °C 30 s; with final extension of 72 °C 5 min. Samples were quantified using Qubit dsDNA HS Assay (Life technologies) before pooling. The sequencing run had on average of 111,536 sequences per sample. Samples were paired and quality trimmed to q20. OTUs were picked at 97% using Open-reference OTU picking strategy (utilizing Greengenes database release Feb-2011; http://greengenes.lbl.gov), and chimera checked with cluster less than 4 sequences removed using scripts in QIIME.

NMDS statistical analysis of all sequencing and the community compositional analysis were undertaken in R statistical package.

All sequencing data has been deposited in the European nucleotide archive and available under accession number PRJEB12833.

### 2.9 [^3^H]NE uptake and release from the caecum

Mice 72 h after sham surgery or experimental stroke were killed by decapitation and the caecum was rapidly removed. Caecum tissues without caecal content were dissected into 2–3 mm pieces, immediately placed into ice-cold Krebs’ solution (113 mM NaCl, 4.7 mM KCl, 1.2 mM MgSO_4_, 2.5 mM CaCl_2_, 25 mM NaHCO_3_, 1.2 mM KH_2_PO_4_, 115 mM glucose, 0.3 mM Na_2_EDTA, and 0.03 mM ascorbic acid), and continuously gassed with a mixture of 95% O_2_ and 5% CO_2_. Caecum slices were washed with 5 ml of Krebs’ solution, and loaded for 45 min with [^3^H]NE at a concentration of 10 μCi in 1 ml of Krebs’ solution. Slices then were washed three times with 10 ml of ice-cold oxygenated Krebs’ solution and transferred into a four-channel microvolume (100 μl) superperfusion system kept at 37 °C (Vizi et al., 1985) superfused with Krebs’ solution at a rate of 0.5 ml min^−1^ for 60 min (preperfusion period), and the effluent was discarded. After preperfusion, 19 × 3 min fractions were collected. Electrical stimulation (20 V; 10 Hz; 2 ms; 1200 impulses) was applied during the third (S_1_) and the thirteenth sample (S_2_). In some experiments tetrodotoxin (3 μM) was added during the ninth sample (after S1) which was present thereafter in the perfusion fluid to investigate its effect on S2-induced NE release. Then, the caecum slices were removed from the chamber and homogenised in 5 ml of 10% trichloroacetic acid. A 0.5 ml aliquot of the supernatant was added to 2 ml of scintillation mixture (Ultima Gold; Packard, Meridian, CT), and the radioactivity was measured with a Packard 1900 TR liquid scintillation counter. Radioactivity was expressed in terms of disintegration per minute per gram of wet tissue (becquerels per gram). The [^3^H]NE uptake of slices was defined as the tissue content of radioactivity at the beginning (*C*_B_) of the perfusion period. This value was calculated according to the following equation: Σ*_i_*_=1–19_ *FR_i_* + *C*_E_ = *C*_B_, where *FR_i_* is the released radioactivity in the fraction number *i*, and *C*_E_ is the tissue content measured at the end of the experiment. The neuronal release of [^3^H]NE was measured by the integration of the surplus release over baseline in response to electrical stimulation.

### 2.10 Histology

72 h after sham surgery or experimental stroke the caecum was removed and placed immediately into 4% paraformaldehyde (PFA, pH = 7.4) for 24 h prior to paraffin embedding. 5 μm thick sections were mounted onto gelatin-coated slides, deparaffinized and stained with haematoxylin and eosin (H&E), Azan or *periodic acid Schiff*-*alcian blue* (PAS), then dehydrated and coverslipped with Depex mounting medium. Mucin-containing cells visualized by PAS staining have been quantified in a blinded manner on 3–3 randomly selected caecum sections in each mouse (n = 4). Catecholaminergic nerve fibers in the caecum were visualized with immunohistochemistry using mouse-anti TH monoclonal antibody (1:100, DiaSorin). The staining was developed with anti-mouse ImmPRESS reagent followed by DAB-Ni peroxidase kit (Vector Laboratories, Burlingame, CA, US), and sections counterstained with cresyl violet.

### 2.11 Statistical analysis

Statistics were undertaken using the R-package, with multivariate analysis was undertaken on these data using the Vegan (Oksanen et al., 2012) and Ecodist (Goslee and Urban, 2007) packages in R. A Non parametric version of multidimensional scaling (NMDS) was used to assess communities using bray-curtis dissimilarities to characterise the difference between communities. The calculated dissimilarity matrix is compressed and modelled in 2 dimensions for NMDS figures in which the physical distances represent the level of similarity between samples, and permutational multivariate analysis of variance using distance matrices (Adonis in Vegan) for comparisons between groups on the distance matrix to give significance values.

## 3 Results

### 3.1 Experimental cerebral ischemia

**Fig. 1:**
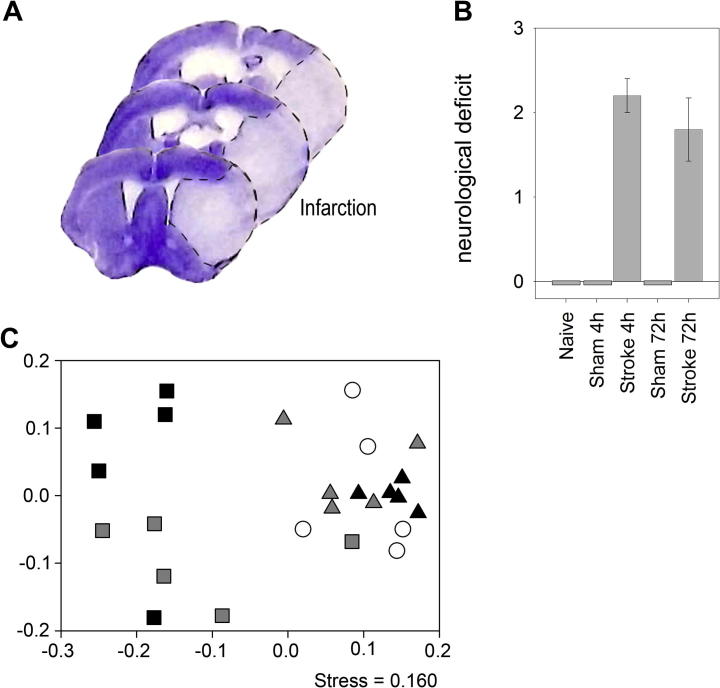
Neurological deficit and bacterial community profiling of brain injury mice and controls by DGGE: (A) Brain injury is shown after 60 min MCAo and 72 h reperfusion as identified by cresyl violet staining, dotted line indicates infarct boarder. (B) Neurological deficit score for mice post surgery significant effect of treatment (ANOVA: F_4,20_ = 33.88, p = <0.01) post hoc TukeyHSD confirmed stroke increase neurological deficit (p < 0.001), with no difference between 4 h or 72 h post brain injury (p = 0.58) Error bars are standard error of the mean, n = 5. (C) NMDS analysis of caecal bacterial communities assessed by DGGE of the 16S rRNA gene, profile of Naïve, Sham and Brain injury mice caecal bacterial communities at t = 4 h and 72 h post operation. Samples are as follows: ○ = t 0; △ = t 4 h; □ = t 72 h post treatment; white = naïve; grey = sham; black = brain injury. Axis represent scale for simularity distance scores between sample centered to (0,0). PERMANOVA: Naïve vs. Sham and Brain injury at 4 h post operation (adonis: F.Model_2,12_ = 1.34, p = 0.159), Naïve vs. Sham 72 h (adonis: F.Model_1,8_ = 3.59, p = 0.016); Naïve vs. Brain Injury 72 h, (adonis: F.Model_1,8_ = 5.90, p = 0.008); Sham 72 h vs. Brain Injury 72 h, (adonis: F.Model_1,8_ = 1.51, pr = 0.17). (For interpretation of the references to colour in this figure legend, the reader is referred to the web version of this article.)

Experimental stroke induced in mice by occlusion of the middle cerebral artery (MCAo) resulted in cerebral ischemia affecting the ipsilateral striatum and the cerebral cortex consistent with our earlier studies (Dénes et al., 2010, Dénes et al., 2011) (Fig.1A). Sensory-motor functional assessment indicated severe neurological deficit in mice that had undergone 60 min MCAo and 4 or 72 h reperfusion, but no deficit was observed in naïve or sham mice (Fig.1B). We then sought to determine the effects of this injury on the intestinal microbiota. Since experimental stroke induced by MCAo requires acute surgical intervention that includes anaesthesia, we set up additional experimental groups to control for the potential effects of surgical stress and the anaesthetic isoflurane.

### 3.2 Characterisation of microbial communities

To determine the effects of experimental stroke on intestinal microbiota we initially used bacterial community profiling of the caecum by DGGE. Caeca were removed and total genomic DNA was extracted for bacterial community profiling by DGGE. Data were analysed using non-metric multidimensional scaling (NMDS). Fig.1C reveals specific changes in the bacterial communities. No significant change was observed between naïve mice, sham mice, or mice that had undergone MCAo at 4 h post operation using PERMANOVA on the distance matrix created. However, at 72 h post operation there was a shift in microbial populations as result of surgery with an indication of the separation of sham and brain injury animals on their community profile (Fig.1C). The caecal microbial community composition in mice that had experimental stroke and 72 h reperfusion had significantly different communities to naïve mice. Sham operated individuals also had a significantly different microbial community to the naïve population (Fig.1C), indicating that sham surgery alone causes a profound change in caecal microbial community structure. To determine whether differences in caecal microbiota between naïve and operated mice were a result of anaesthesia rather than surgery, we analysed the caecal microbiota in animals that had received anaesthetic alone. Caeca were sampled 72 h post anaesthesia, and from control mice, and community profiled as described above by DGGE (data not shown). NMDS analysis demonstrated that there was no significant difference between mice as a result of the anaesthetic (S1 Figure, adonis: F.Model_1,9_ = 1.83, pr = 0.081). Therefore the changes identified in the sham mice were mostly a result of the surgery, not the anaesthetic with the small sample (n = 5–6) tested.

To characterise the specific changes occurring as a result of brain injury the composition of the caecal microbiota were determined by 16S rRNA gene amplification followed by pyrosequencing generating an average of 12,335 sequences per sample. Samples demonstrated deep coverage by the levelling of rarefaction curves (S2 Figure) and using goods coverage estimate (Good, 1953) we found on average 98.99% coverage of operational taxanomic units in samples (OTUs: bacterial species defined at the level of >97% similarity on sequence level of the 16S rRNA gene).

**Fig. 2:**
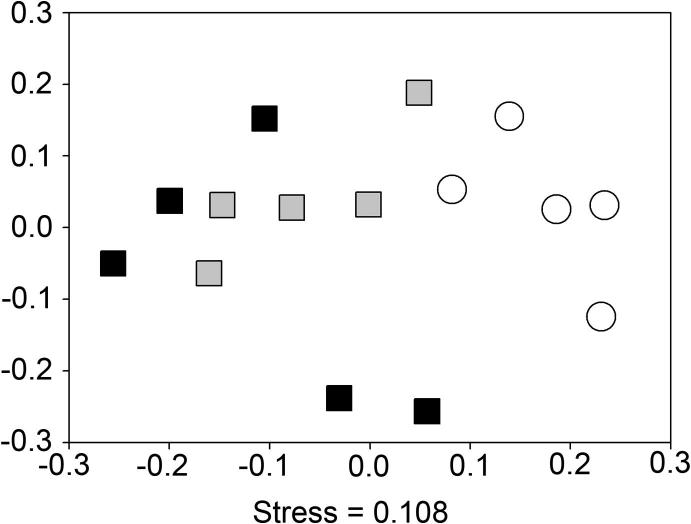
16S amplicon pyrosequencing analysis of mouse caecum from niave, sham and brain injury mice. Bacterial species relative abundances from rarefied OTU data (1866 sequences) were used for NMDS analysis Samples are as follows: ○ = t 0; □ = t 72 h post treatment; white = naïve; grey = sham; black = brain injury. Axis represent scale for simularity distance scores between sample centered to (0,0). Pair-wise comparisons were undertaken to identify significant differences using PERMANOVA. Naïve vs. Sham (adonis: F.Model_1,8_ = 2.12, p = 0.008); Naïve vs. Brain Injury (adonis: F.Model_1,8_ = 2.24, p = 0.008); Sham vs. Brain injury, (adonis: F.Model_1,8_ = 1.02, p = 0.484).

**Fig. 3:**
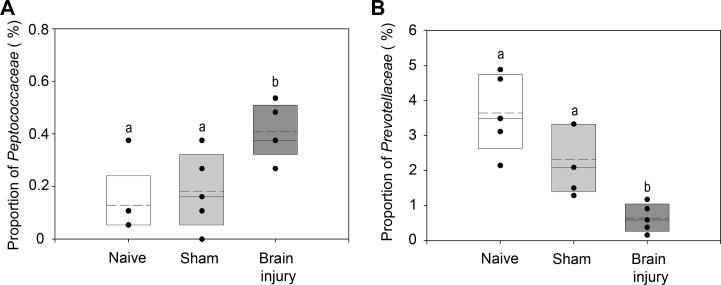
Proportions of bacterial taxa that were identified to change significantly as a result of experimental stroke or sham surgery. ANOVA was undertaken on all taxa identified with FDR correction of P-values. (A) *Peptococcaceae* proportions of community between naïve, sham, and brain injury mice were significantly different (p-adjust = 0.026). with labels a and b denoting treatments significantly different (p < 0.05) identified using TukeyHSD posthoc test, (B) *Prevotellaceae* proportions of community between naïve, sham, and brain injury mice were significantly different (p-adjust = 0.019) with labels a and b denoting treatments significantly different (p ⩽ 0.05) identified using TukeyHSD posthoc test. Upper and lower limits of box represent 75th and 25th percentile, solid line in median, dotted line mean. Dots represent actual values for each mouse.

Assessment of the Shannon diversity index (Shannon, 1948) of the sequence data showed that there were no significant differences between samples (ANOVA: F_2,12_ = 0.50, p = 0.62). However, NMDS analysis identified that there was a significant shift in communities as result of stroke or surgery (Fig. 2). To identify any specific bacterial taxa that change as a result of brain injury alone the rarefied sequence data was analysed using proportional data for each taxa calculated using QIIME. ANOVA tests were undertaken for each taxa to identify if treatment had a significant effect on their proportion. To account for any increases in type I errors, the resulting p values table was corrected using the commonly used FDR correction which is commonly used for this type of analysis (Benjamini and Hochberg, 1995). We identified that brain injury had a significant effect on the proportion of Peptococcaceae (Fig.3A). TukeyHSD posthoc tests confirmed that as a result of brain injury there was a significant increase in the proportion of Peptococcaceae in comparison to Naïve and Sham mice (p < 0.05). The proportions of Prevotellaceae decreased as a result of either treatment (Fig.3B). TukeyHSD posthoc tests identified that there was a decrease in the proportions of Prevotellaceae as a result of sham in comparison to naïve although not significant (p = 0.08), which declined further as a result of stroke (p ⩽ 0.05). Therefore these data establish that there are specific significant changes to the caecal microbiota as a consequence of experimental stroke that cannot be attributed to the effects of surgery alone.

To assess whether changes as a result of brain injury could occur by chance variation in microbial communities, another experiment was undertaken where 10 naïve mice (n = 5 per cage) were monitored by stool sampling at 0, 28, and 41 days, with the samples subjected to 454 pyrosequencing as detailed above. No significant effect of time or cage housed was found in Shannon bacterial diversity (ANOVA: F_2,16_ = 0.40, p = 0.676) and (ANOVA: F_1,8_ = 1.46, p *=* 0.261) respectively (S3 Figure). NMDS analysis of bacterial composition (S4 Figure), identified a small time dependent change on the bacterial community composition (adonis: F.Model_1,28_ = 1.71, p = 0.022), which would not be unexpected over 41 days. ANOVA with FDR correction did not identify any taxa that changed with time between samples. This would indicate that microbial communities are very stable in C57BL/6 mice caged in our animal facility, giving us confidence in the brain injury effects identified.

### 3.3 Linking bacterial changes to functional outcome after stroke

**Fig. 4:**
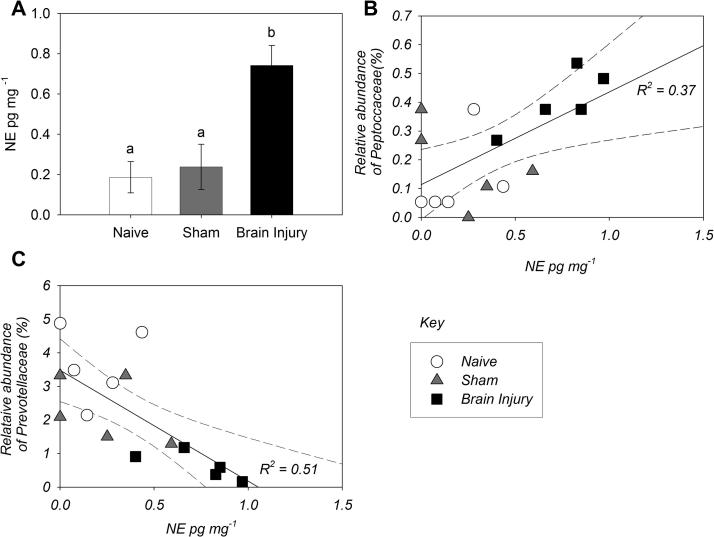
Intestinal microbiota changes correlate with gut noradrenaline (NE) levels (A) Mice that had undergone 60 min MCAo and 72 h reperfusion show significantly increased NE levels in gut tissue homogenates (ANOVA: F_2,12_ = 10.02, p = 0.003; Posthoc TukeyHSD significance <0.001, with the labels a and b denoting samples significantly different, error bars are standard error of the mean, n = 5. (B) Peptococcaceae relative abundance correlate with intestinal NE levels (Regression: R^2^ = 0.370, F_1,13_ = 7.56, p = 0.02), Equation of line y = 0.322 * x + 0.114. (C) Prevotellaceae decrease in the intestinal proportionally to increasing NE levels after injury (Regression: R^2^ = 0.512, F_1,13_ = 13.58, p = 0.003), Equation of line y = −3.299 * x + 3.480. Samples are as follows: ○ = Naïve; △ = Sham 72 h post treatment; □ = Brain injury 72 h post treatment.

To investigate whether brain injury drives autonomic changes in the gut, we assessed levels of key neurotransmitters in the tissue of the caecum. Levels of adrenaline, serotonin, and substance P in the caecum did not change (S4 Figure), but noradrenaline (NE) was significantly increased 72 h after experimental stroke (Fig.4A). Increased NE levels positively correlated with neurological deficit scores (S5 Figure). Relative abundance of Peptococcaceae in the caecum showed a significant positive correlation, whereas abundance of Prevotellaceae showed a significant negative correlation with NE levels (Fig.4B &C) and neurological deficit demonstrated the same correlations (S5 Figure). We have also tested changes in several inflammatory markers in the intestinal tissue, which are known to be associated with alterations in the microbiota. Of these markers RANTES (CCL5) levels showed a positive correlation with the relative abundance of Peptococcaceae in the caecum (S6 Figure).

### 3.4 Brain injury leads to increased release of NE from sympathetic nerves in the caecum and altered mucoprotein production

**Fig. 5:**
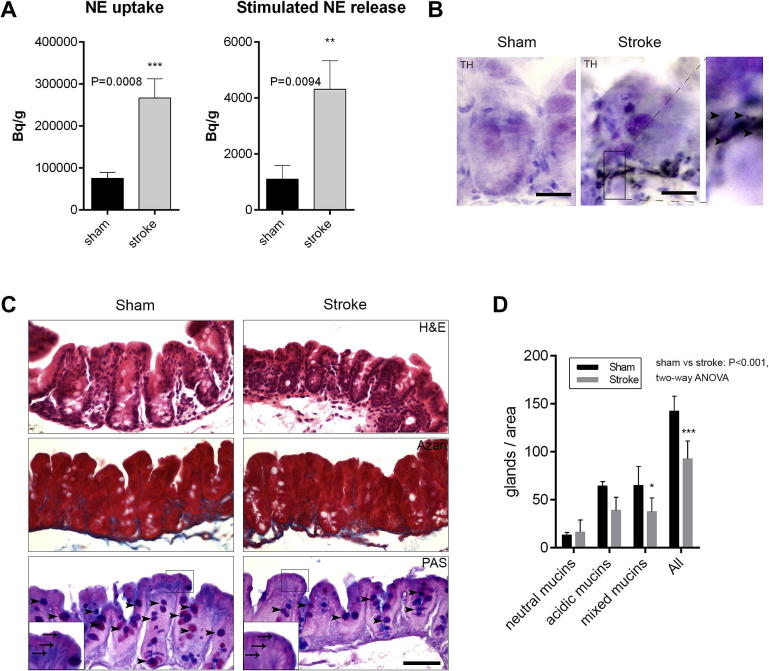
Brain injury results in altered noradrenaline (NE) release and mucoprotein production in the caecum. (A) Tissue uptake and stimulated release of ^3^H-NE was significantly increased in the caecum 72 h after experimental stroke (^**^P < 0.01, ^***^P < 0.0001, unpaired *t* test), error bars are standard error of the mean, n = 6–8). (B) Tyrosine hydroxilase immunopositive nerve fibers (arrowheads) are most abundant at the basis of the intestinal epithelium found often in the proximity of goblet cells in mice 72 h after brain injury (cresyl violet counterstain). (C) Haematoxylin and eosin (H&E), Azan and *periodic acid-Schiff-alcian blue* (PAS) staining of paraffin-embedded caecum sections is shown, 72 h after sham surgery or experimental stroke. PAS staining identifies mucoprotein-containing cells (magenta – neutral mucins, blue – acidic mucins, magenta/blue –mixed mucins) and indicates less mucoproteins associated with the apical part of intestinal epithelial cells (shown by arrowheads on insert). (D) Quantification of PAS staining (C). Brain injury is associated with less mucin-containing cells in the caecum compared to sham animals (^*^P < 0.05, ^***^P < 0.0001, one-way ANOVA followed by TukeyHSD posthoc test), error bars are standard error of the mean, n = 4. Scale bars: B – 50 μm; C – 100 μm. (For interpretation of the references to colour in this figure legend, the reader is referred to the web version of this article.)

To investigate how brain injury leads to increased NE production in the caecum, we used a well-established *ex vivo* approach allowing selective assessment of noradrenergic autonomic regulation in the tissue. Caecum tissue blocks isolated 72 h after sham surgery or experimental stroke were rinsed carefully to eliminate caecal content and were incubated with ^3^H-NE, allowing uptake and release of ^3^H-NE upon electric stimulation. Brain injury resulted in a profound increase in autonomic outflow in the caecum indicated by an over threefold increase in ^3^H-NE tissue uptake and release induced by a 10 Hz stimulation (Fig.5A). To further confirm that brain injury altered autonomic nerve function in the gut and increased NE production/release (Fig.5A) is not due to other cells, such as macrophages that can produce catecholamines (Nguyen et al., 2011), tetrodotoxin was used that selectively blocks the firing of action potentials in nerves by inhibiting voltage-gated sodium channels (Vizi et al., 1995). Tetrodotoxin completely prevented stimulation-evoked ^3^H-NE release in the caecum (S7 Figure), indicating that brain injury increases noradrenergic autonomic outflow in the caecum. Histology also confirmed that nerves containing tyrosine hydroxylase (TH, a rate-limiting enzyme of catecholamine biosynthesis) were more abundant in the caecum of mice 72 h after brain injury compared to sham animals (Fig.5B). TH-positive nerve fibers and nerve endings were mostly found in close proximity to mucoprotein-containing cells at the basis of the intestinal epithelium (Fig.5B). To test whether the increased levels of NE were directly affecting the growth of Peptococcaceae and Prevotellaceae in the caecum, caecal contents were obtained from naive mice and cultured anaerobically for 24 h with increased levels of NE. Analysis by Q-PCR showed no correlation between the level of NE and the growth of both bacterial genera (S8 Figure). As such it is our hypothesis that the effects of increased levels of NE on the Peptococcaceae and Prevotellaceae are indirect. It is known that autonomic regulation could indirectly influence the gut microbiota via altered goblet cell function (Kim and Ho, 2010). To test whether brain injury had any effect on mucoprotein production in the caecum the number of cells in the intestinal mucosa containing mixed (both acidic and neutral) mucoproteins and the total number of goblet cells was counted. Both the number of cells containing mixed mucoproteins and the total number of goblet cells were significantly reduced 72 h after brain injury compared to sham mice (Fig. 5C and D). However, no sign of local tissue inflammation, leukocyte infiltration, fibrosis or tissue lesions were seen in the caecum after brain injury as assessed on haematoxylin and eosin (H&E)- and Azan-stained caecum sections (Fig.5C).

### 3.5 Manipulation of the sympathetic nervous system leads to altered mucoprotein production, bacterial community changes, and reduced Prevotellaceae levels in the caecum

**Fig. 6:**
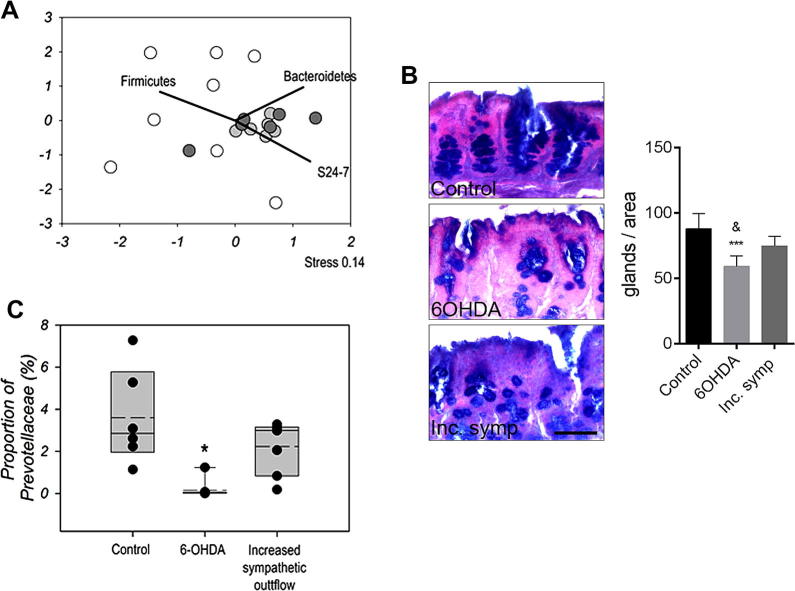
Impact of manipulations of the sympathetic nervous system. (A) Impact on community microbiota assessed by NMDS analysis of 16S amplicon illumina sequencing relative abundances from rarefied OTU data (36,904 sequences). Controls = white, 6-OHDA treated = Light grey, Increased systemic sympathetic autonomic tone = dark grey. 6-OHDA samples were significantly different to controls (adonis F.model_1,12_ = 2.90, p = 0.0012), whereas Increased systemic sympathetic autonomic tone had no impact. Significant correlations were identified with the Phyla of Firmicutes (R^2^ = 0.60, p = 0.008), Bacteroidetes (R^2^ = 0.58, p = 0.01) and the Bacteroidetes family S24-7 (R^2^ = 0.78, p = 0.017). (B) *Periodic acid-Schiff-alcian blue* (PAS) staining of paraffin-embedded caecum sections is shown in control mice and after 6-OHDA administration or atomoxetine/yohimbine treatment that leads to mild increase in sympathetic tone (Inc. symp). Number of PAS-positive goblet cells is significantly reduced 72 h after 6-OHDA compared to Control (p < 0.001) and Inc. symp. (^&^p < 0.05) mice. Scale bar: 100 μm. Error bars are standard error of the mean, n = 5–6. (C) Significant shifts (^∗^) in the proportions of Prevotellaceae as a result of 6-OHDA treatment p < 0.05. Upper and lower limits of box represent 75th and 25th percentile, solid line in median, dotted line mean. Dots represent actual values for each mouse.

To investigate whether changes in sympathetic activity lead to similar alterations in the caecal microbiota *in vivo* to those seen after ischemic brain injury, two different pharmacological approaches were used 1) A mild increase in systemic sympathetic autonomic tone induced by daily administration of atomoxetine (a NE reuptake inhibitor) and yohimbine (an α_2_-adrenergic receptor antagonist) or 2) intraperitoneal administration of 6-hydroxydopamine (6-OHDA), which leads to robust NE release followed by depletion of noradrenergic nerve terminals in peripheral tissues. Impact on microbial communities was assessed by sequencing of 16 rRNA gene amplicons using illumina Mi-seq platform resulting in an average of 111,536 sequences per sample with greater than 99.8% coverage of OTUs estimated by good coverage estimates (Good, 1953). A rarefied subset of 36,904 sequences was used for all subsequent analysis. The mild increase in systemic sympathetic autonomic tone had no effect on microbial communities in the caecum 72 h later, as identified by 16S rRNA sequencing (Fig. 6A). In contrast, the administration of 6-OHDA resulted in profound changes in the gut microbiota (Fig.6A). The 6-OHDA treatment resulted in differential perturbations of the microbial community causing a significant spread and separation from the control mice when assessed by NMDS (adonis F.model_1,12_ = 2.90, p = 0.0012). Correlation of bacterial abundances in relation to the NMDS plot identified significant correlations with the Phyla of Firmicutes (R^2^ = 0.60, p = 0.008) and Bacteroidetes (R^2^ = 0.58, p = 0.01) and the Bacteroidetes family S24-7 (R^2^ = 0.78, p = 0.017) (Fig.6A) populations indicating dramatic and differential shifts in the populations. 6-OHDA treatment significantly decreased goblet cell numbers in the caecum, whilst atomoxetine/yohimbine treatment had no effect (Fig.6B). Moreover, 6-OHDA treatment specifically resulted in a 20-fold reduction in Prevotellaceae levels in the caecum, a reduction also observed after experimental stroke (Fig.6C).

### 3.6 Severity of traumatic brain injury correlates with changes in the gut microbiota

**Fig. 7:**
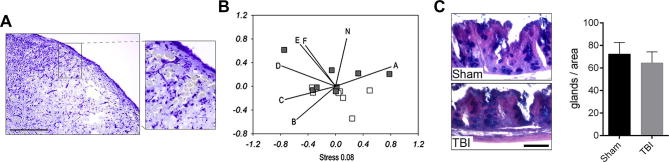
Impact of traumatic brain injury (TBI) on microbiota. (A) Cresyl violet staining indicates brain injury induced by TBI in the cerebral cortex. Scale bar: 500 μm. (B) Impact on community microbiota as a result of TBI assessed by NMDS analysis of 16S amplicon Ilumina sequencing. Rarefied OTU data (36,904 sequences) was used with samples as follows: Controls = white, TBI = dark grey. A strong correlation between neurological deficit and community compositions was seen label N (R^2^ = 0.6797 p = 0.0013). Other significant correlations explaining the shifts in NMDS were identified and marked with the letters as follows (A) Clostridiales (R^2^ = 0.76 p = 0.015), (B) Bacteroidetes (R^2^ = 0.63 p = 0.02), (C) α-proteobacteria (R^2^ = 0.59 p = 0.039), (D) Proteobacteria (R^2^ = 0.73 p = 0.008), (E) Cyanobacteria (R^2^ = 0.76 p = 0.002), (F) Porphyromonadaceae (R^2^ = 0.66 p = 0.027). (C) *Periodic acid-Schiff-alcian blue* (PAS) staining of paraffin-embedded caecum sections is shown 72 h after sham surgery or traumatic brain injury (TBI). Scale bar: 100 μm. Error bars are standard error of the mean, n = 7–8. (For interpretation of the references to colour in this figure legend, the reader is referred to the web version of this article.)

We next investigated the impact of alternative models of brain injury on the gut microbiota, and thus subjected mice to mild traumatic brain injury (TBI) using a closed-head injury model (Fig.7A). As with the manipulation of the sympathetic nervous system, communities were assessed using illumina Mi-Seq multiplexed with the run described above. The severity of injury from TBI, as with ischemic brain injury described above, correlated with changes in caecal microbiota (Fig.7B). Bacterial population shifts on NMDS that indicate correlations with neurological deficit as a result of TBI, were identified in Bacteroidetes, the Bacteroidetes family Porphyromonadaceae, Firmicutes and α-Proteobacteria. However in contrast to ischemic brain injury, TBI did not result in changes in Peptococcaceae and Prevotellaceae. Correspondingly, goblet cell numbers after TBI were identical to that seen in control mice (Fig.7C), suggesting that different forms of brain injury shape changes in the gut microbiota through multiple mechanisms.

## 4 Discussion

There is an increasing appreciation of the importance of the intestinal microbiota and its contribution to the development and maintenance of physiological systems and homeostasis. Altered intestinal microbiota are linked to neuro-developmental disorders (Kang et al., 2013), in addition to a number of immune and inflammatory disease states (Flint et al., 2012, Wu, 2011, Spor et al., 2011, Abt et al., 2012, Ganal et al., 2012, Ayres et al., 2012, Dupont and Dupont, 2011, Le Chatelier et al., 2013, Yoshimoto et al., 2013). While a number of factors are known to influence the intestinal microbiota, such as diet, age, and disease, the effects of brain function per se have not been studied. We show that impairment in brain function, induced by experimental stroke, caused specific and significant changes in the caecal microbiota. These stroke-specific changes occur relatively rapidly, within 72 h, and involve a significant decrease in the levels of Prevotellaceae and an increase in the levels of Peptococcaceae. Prevotellaceae have been shown to be part of the core microbiota of C57Bl/6 mice (Gu et al., 2013), as such the consequences of reducing the prevalence of Prevotellaceae is likely to be significant. In humans Prevotellaceae are associated with agrarian diets rich in plant derived material and reductions in the abundance of Prevotellaceae have been detected in the microbiota of autistic children (Luczynski, 2016), Crohn’s disease (Prindiville et al., 2004) and in children suffering from type 1 diabetes (Mejía-León et al., 2014). In contrast, abnormal increases in Prevotellaceae has been found to exacerbate DSS-induced colitis in mice (Elinav et al., 2011). The significance of the increase in the abundance of Peptococcaceae is less clear since little is known about their role in the intestinal microbiota. Peptococcaceae are relatively minor components of the intestinal microbiota of both mice and humans, and the changes we detect here may be a consequence of the decrease in the levels of Provetellaceae. Nevertheless, since only brain injury, but not surgical stress resulted in changes (over 3-fold) in Peptococcaceae, these changes could be specific indicators of brain injury and their predictive value for functional outcome should be tested in patients with various forms of brain injury. Interestingly, the proinflammatory cytokine, CCL5 (RANTES), which we found to positively correlate with Peptococcaceae levels, has been linked with the development of colitis (Elinav et al., 2011), therefore the functional role of RANTES in stroke-induced gut microbiota changes will need to be investigated in future studies.

These changes in the caecal microbiota were correlated both with increased levels of NE and noradrenergic innervation as well as the severity of injury. It is well known that NE levels increase following trauma or injury (Millen et al., 1985, Woolf et al., 1992, Lyte and Bailey, 1997) and stroke is known to induce NE in the circulation in line with increased sympathetic activity in both humans and experimental animals (Myers et al., 1981, Cechetto et al., 1989). However this is the first evidence for increased NE release and noradrenergic innervation in the caecum following stroke. It is known that NE can be sensed and utilised by bacteria in caecal microbiota and that NE-like molecules can be synthesised by bacteria (Karavolos et al., 2013, Asano et al., 2012, Lyte, 2004). However the anatomical and neurochemical evidence data presented here demonstrate the source of the NE in the caecum following stroke would appear to be host derived from sympathetic innervation. It is currently unclear whether brain injury-induced increases in sympathetic outflow in the caecum are due to direct actions of altered central sympatho-motor responses or could also be influenced by changes in parasympathetic activity mediated by the vagus nerve. However these data suggest that specific brain-induced changes in gut NE will influence the microbiome-gut-brain axis and thus affect outcome and behaviour. The effect of early life stress on microbiota has been demonstrated previously, suggesting that stress-induced central or peripheral events could alter the intestinal microbiota (O’Mahony et al., 2009).

Gastrointestinal dysfunction occurs frequently in stroke patients in the form of altered intestinal motility, abdominal pain, gastric distension, constipation or ulcers that are linked to altered autonomic activity (Korpelainen et al., 1999, Su et al., 2009, Bracci et al., 2007, Hilz et al., 2011). Increasing stroke severity is associated with progressive loss of overall autonomic modulation and a progressive shift toward sympathetic dominance in stroke patients (Hilz et al., 2011). These data therefore suggest that a specific neuronal input or an imbalance in neuro and inflammatory mediators (such as NE reported here for example) in the interstitial intestinal tissue contribute to the signals that regulate the structure of the intestinal microbiota. Although it is known that the growth in pure culture of *Prevotella intermedia* is inhibited by the presence of stress hormones such as NE (Jentsch et al., 2013), when the mouse microbiota was cultured *in vitro* in increasing concentrations of NE we did not detect any difference on the levels of Prevotellaceae or Peptococcaceae (S8 Figure). This difference may well reflect that in our experiments we were studying the growth of caecal microbiota consisting of many interacting bacterial species, an approach more likely to reproduce effects inside the caecum rather than pure culture. As such the increase in host-derived NE seen in the experimental stroke may be indirectly affecting the relative growth of Prevotellaceae and Peptococcaceae within the caecal microbiota resulting their changes in abundance. Pharmacological manipulation of the peripheral autonomic nervous system by 6-OHDA strengthened our conclusions that a decrease in Prevotellaceae in the caecum seen after experimental stroke could be due to altered autonomic activity. Intraperitoneally administered 6-OHDA does not cross the BBB (Williams et al., 1981) and leads to rapid release of NE in the periphery, which has been shown to lead to immediate changes in diverse bacterial species (Lyte, 2014, Pullinger et al., 2010). This is followed by depletion of noradrenergic terminals. We found decreased Prevotellaceae levels 72 h after 6-OHDA administration, which was similar to what seen after experimental stroke. It is currently unclear whether ischemic brain injury leads to increased sympathetic outflow in the caecum via increasing central autonomic tone, or whether cessation of central autonomic control after brain injury is compensated by local increases in NE in the gut. Nevertheless, increased NE release or the dysregulation of autonomic control of the gut after both ischemic brain injury and 6-OHDA would explain similar changes seen in Prevotellaceae.

It is known that autonomic regulation can effect goblet cell function (Kim and Ho, 2010). As such NE could indirectly influence the gut microbiota via altered goblet cell function (Kim and Ho, 2010). Analysis of caecal tissue from stroke animals demonstrated that the number of cells containing mixed mucoproteins and the total number of goblet cells were significantly reduced 72 h after brain injury compared to sham mice (Fig.5C and D). As such changes to the mucosal surface are likely to be generated as a consequence of brain injury. Changes in mucin levels have been linked to gut health and susceptibility to infection (Kim and Ho, 2010), of which bacteria from the family Prevotellaceae have been demonstrated to colonise and utilise mucin (Wright et al., 2000). The autonomic nervous system is also known to dampen immune responses (Reyes-García and García-Tamayo, 2009) although here no sign of local tissue inflammation, leukocyte infiltration, fibrosis or tissue lesions were seen in the caecum after brain injury as assessed on haematoxylin and eosin (H&E)- and Azan-stained caecum sections (Fig.5C). Importantly, 6-OHDA treatment resulted in reduced number of goblet cells in the caecum similarly to what was found after experimental stroke and a decrease in Prevotellaceae was observed in both cases. In contrast, traumatic brain injury did not alter goblet cell numbers or Prevotellaceae levels in the caecum, and caused different community changes.

It should be noted that an important observation of our studies was the effect of sham surgery on the caecal microbiota. We have previously reported the surprisingly significant effects of sham surgery on levels of inflammatory mediators in peripheral tissues and the circulation (Denes et al., 2011). In the closed head model of mild traumatic brain injury used here, sham mice only experienced a relatively small manipulation (a small cut on the skin covering the skull) compared to the sham surgery required for the MCAo model of experimental stroke, whilst brain injury and functional deficit were much smaller in the TBI model. This could explain why TBI did not lead to profound changes in goblet cell function and a decrease in Prevotellaceae in the gut, whilst a correlation between increasing neurological deficit and specific changes in microbiota were found. Thus, microbiota changes after TBI could be in part due to a generic stress response that follows tissue injury and also to a different form of brain injury compared to that seen after experimental stroke. Whether the changes we observe on the intestinal microbiota in response to sham surgery and levels of systemic inflammation reported are functionally linked is unknown. However the implications of these observations are far reaching. Firstly, these data indicate that any surgical intervention may profoundly and rapidly influence the caecal microbiota. Such changes could have effects on post-surgical outcome, particularly if the dysbiosis (microbial imbalance on or inside the body) results in changes that favour the growth of potential opportunistic pathogens within the host microbiota. Secondly, the interpretations of experimental observations made from models that utilise surgical intervention may be confounded by surgery-induced changes in the caecal microbiota and inflammatory status. Thus surgical intervention essentially changes the organisms’ baseline and could in effect create a co-morbid state.

In conclusion, our results are the first to show specific changes in the microbiota due to a change in brain function, and also to surgical stress following tissue injury, with the likely involvement of the autonomic nervous system and goblet cells under certain conditions. Identification of the mechanisms involved in this dysbiosis could help us understand how the connectivity between brain function and the intestinal microbiota contributes to health and disease and have important implications in the treatment of patients following traumatic brain injury.
