# Supplementary material for: Brain injury induces specific changes in the caecal microbiota of mice via altered autonomic activity and mucoprotein production
Source: Brain Behav Immun. 2016 Oct;57:10–20. doi: 10.1016/j.bbi.2016.04.003 (PMC5021180; doi:10.1016/j.bbi.2016.04.003)
Supplement: Supplementary data 1 [file mmc1.docx]

Supporting Information

.
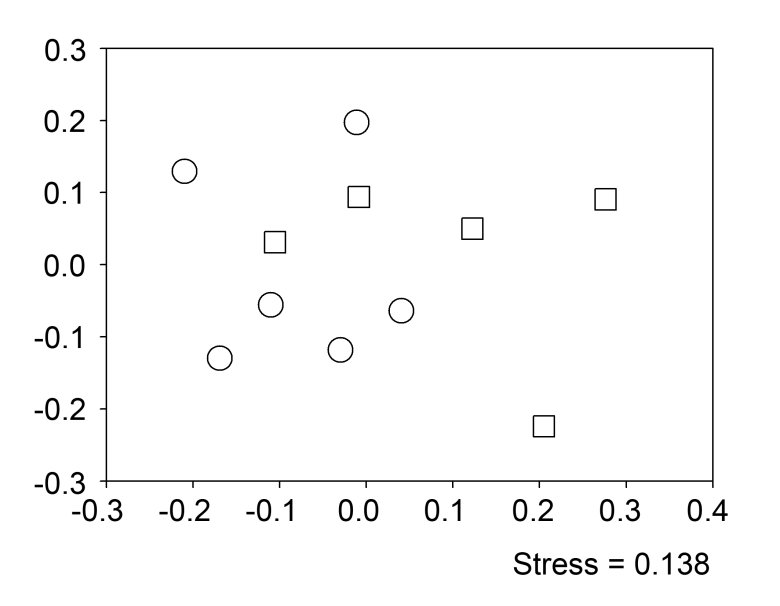


**S1 Figure)** **Impact of Isoflurane on mouse Caecal microbial communities assessed by DGGE and NMDS:** ⭘ = Naïve ; 🞎= Isoflurane treated. Axis represent scale for simularity distance scores between sample centered to (0,0). PERMANOVA demonstrated no significant effect of isoflurane in comparison to naïve mice (adonis: F.Model_1,9_ =1.83, p *=* 0.081).


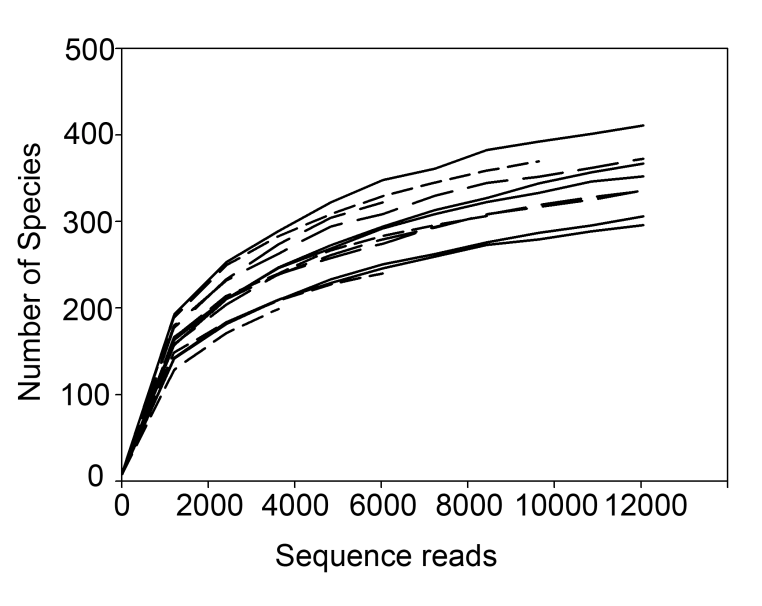


**S2 Figure) 16S amplicon pyrosequencing analysis of mouse caecum from naive, sham and brain injury mice**. Rarefaction curves of sequencing effort against species identified; Species were defined at the 97 % identify level. Samples are as follows; Solid line = naïve mice, long dashed = sham mice, short dashed = brain injury mice


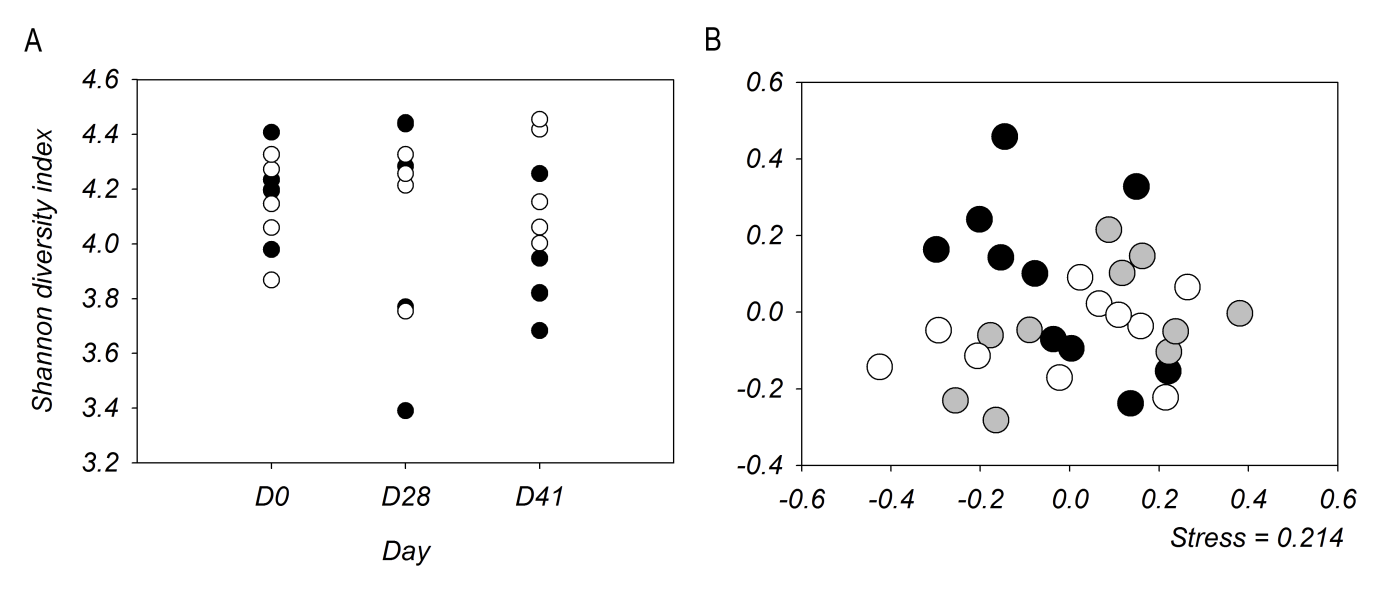


**S3 Figure Analysis of Naïve community stability by pyrosequencing a)** Bacterial community diversity assessed in 10 male C57BL/6 mice over a 41 day period via stool sampling. Diversity was assessed by 454 amplicon pyrosequencing using the 16S rRNA gene. A ratified OTU table to 6480 sequences was used to calculate the Shannon Diversity Index. No significant affects of time (ANOVA: F_2,16_ = 0.40, p *=* 0.676) or cage (ANOVA: F_1,8_ = 1.46, p = 0.261) were identified of bacterial diversity in mice using ANOVA with repeated measures. Colour of dots refer to the cage in which mice were housed. **b**) Non metric multi-dimensional scaling analysis of bacterial community composition was assessed in 10 male C57BL/6 mice over a 41 day period Weighted NMDS analysis was undertaken using proportions of community composition. Stress = 0.214. Axis represent scale for simularity distance scores between sample centered to (0,0). Permutation multivariate analysis of variance with repeated measures was undertaken using adonis (r-package). A small but significant effect of time was identified in community profiles (adonis: F.Model_1,28_ = 1.71, p = 0.022). ⚫ = d0, ⚫ = d28, ⭘ = d41.

**.**

**
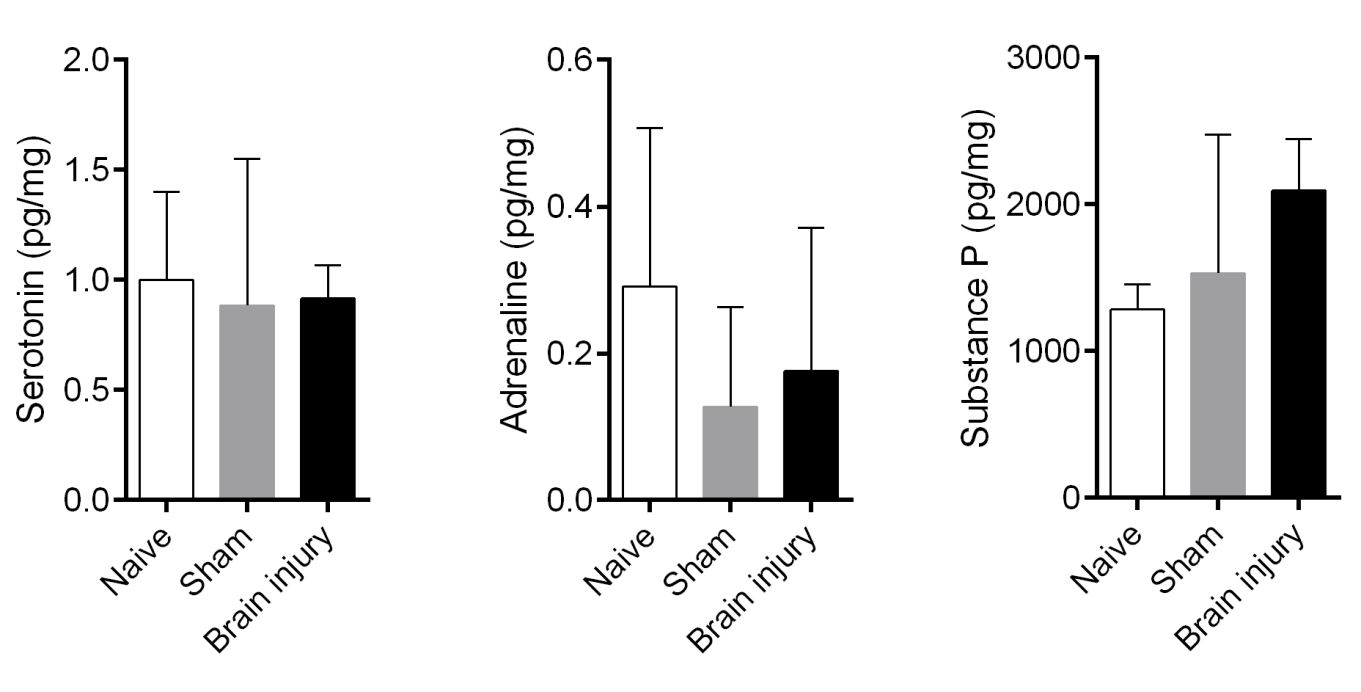
**

**S4 Figure) Serotonin, adrenaline and substance P were measured by ELISA and levels corrected for protein**. Data from caecum tissue homogenates are shown from naïve mice and animals 72 h after sham surgery or experimental stroke, no significant differences were identified. Error bars are standard error of the mean.


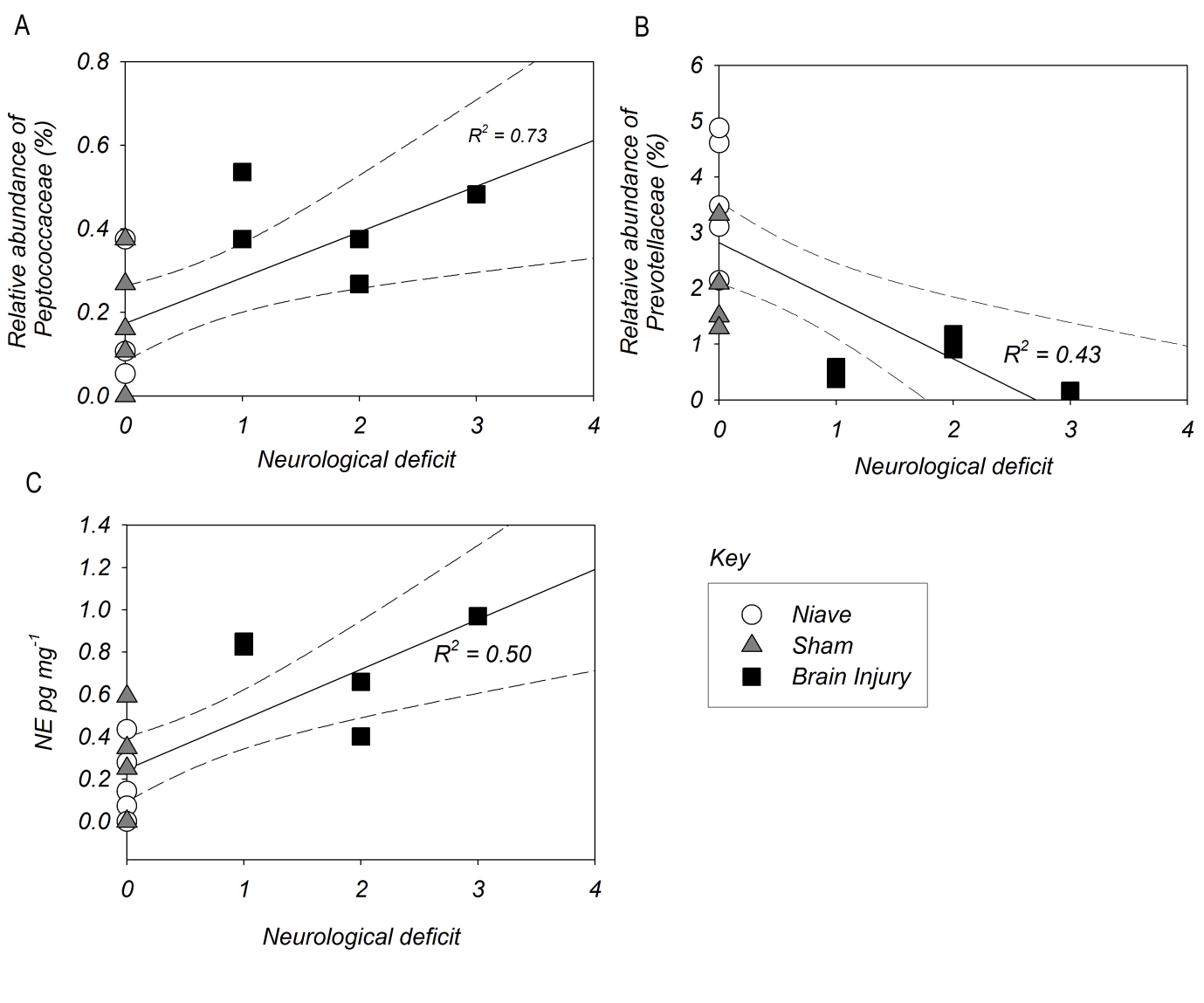


**S5 Figure) Changes in gut microbiota after injury show correlation with neurological outcome a**) Relative abundance of Peptococcaceae is increased in mice with more severe brain injury (Regression: R^2^ = 0.730, F_1,13_ = 35.12, p = <0.001), Equation of line y = 0.182*x + 0.154, dotted line = 95 % confidence interval whereas worse neurological function is associated with decreased Prevotellaceae **b)** levels in the gut (Regression: R^2^ = 0.428, F_1,13_ = 9.73, p = 0.008), Equation of line y = -1.089*x + 2.904. Samples are as follows: ⭘ = Naïve ; Δ = Sham 72h post treatment; 🞎= Brain injury 72 h post treatment. c) Correlation of noradrenaline with neurological deficit, (Regression: R^2^ = 0.503, F_1,13_ = 13.17, p = 0.003), Equation of line y = 0.236*x + 0.247


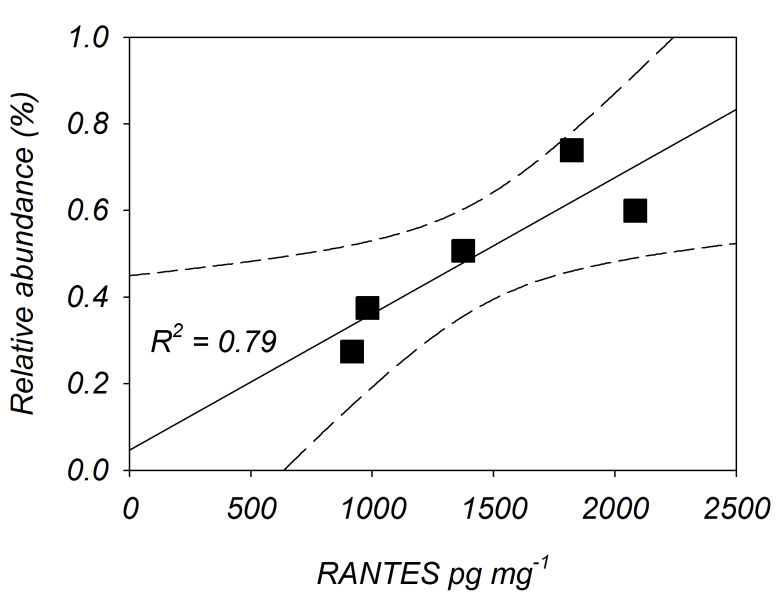


**S6 Figure) Rantes levels in the gut positively correlate with intestinal flora changes.** In mice showing impaired neurological function after 60min MCAo and 72h reperfusion, RANTES (CCL5) tissue levels correlate with relative abundance of Peptococcaceae (Regression: R^2^ = 0.778, F_1,2_ = 10.53, p = 0.047), Equation of line y = 0.0000314*x + 0.0467


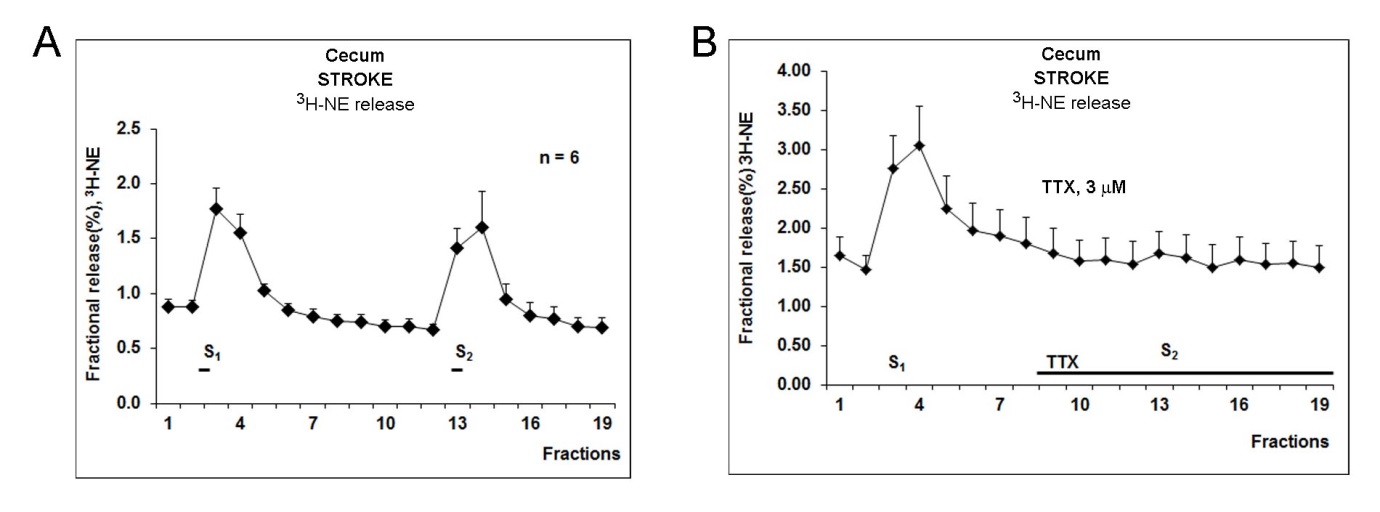


**Figure S7) Release of ^3^H-Noradrenaline from the cecum ex vivo. a)** Electrical stimulation (S1 and S2)-evoked fractional ^3^H-Noradrenaline (NE) release from the cecum isolated 72h after experimental stroke. **b)** Tetrodotoxin (TTX, 3 μM) completely prevented release of ^3^H-Noradrenaline from the cecum (Tetrodotoxin applied prior to the second stimulus, S2). n=6-8.

**
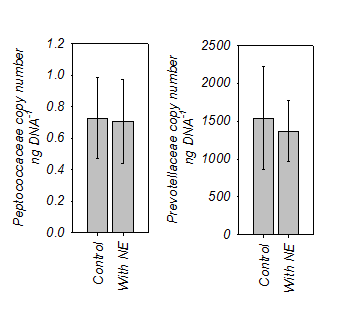
**

**Figure S8) Impact of NE release of growth of Peptococcaceae and Prevotellaceae.** To assess the impact of NE release on the growth of Peptococcaceae or Prevotellaceae populations in the caecum, *in vitro* experiments were undertaken. Caecal content of 3 naïve C57BL/6 mice was surgically removed and rapidly ( <15 mins) put into anaerobic conditions (0.1% Oxygen) and resuspended in Brucella broth media supplemented with hemin (0.5 ug ml^-1^) and vitamin K (0.5 ug ml^-1^) which is suitable for growth of most fastidious and non fastidious anaerobic microorganims found in the gut. This resuspension was diluted 1:100 into media with or without NE at 100 µM. Bacteria were then incubated for 24 h anaerobically, sampled, DNA extracted and populations quantified by qPCR using primers specific to Peptococcaceae (Gitiafroz, R (2012) Thesis, University of Toronto) or Prevotellaceae (Matsuki, T *et al* (2002) AEM 68:5445). t-test analysis demonstrated there was no difference in the Peptococcaceae ( p = 0.39) or Prevotellceae (p =0.67) populations as a result of NE addition. Error bars are standard error of the mean.

**Table S1): Roche Multiplex Identifiers used in 454 pyrosequencing.**

MID ID Barcode sequence

MID-1: CGTCTAGTAC

MID-2: TCTACGTAGC

MID-3: TGTACTACTC

MID-4: ACGACTACAG

MID-5: CGTAGACTAG

MID-6: TACGAGTATG

MID-7: TACTCTCGTG

MID-8: TAGAGACGAG

MID-9: TCGTCGCTCG

MID-10: ACATACGCGT

MID-11: ACGCGAGTAT

MID-12: ACTACTATGT

MID-13: ACTGTACAGT

MID-14: AGACTATACT

MID-15: AGCGTCGTCT
